# Chondroitin sulfate modification of CSPG4 regulates the maintenance and differentiation of glioma-initiating cells *via* integrin-associated signaling

**DOI:** 10.1016/j.jbc.2024.105706

**Published:** 2024-02-02

**Authors:** Akiko Niibori-Nambu, Yoshimune Yamasaki, Daiki Kobayashi, Kiyohiko Angata, Atsushi Kuno, Orasa Panawan, Atit Silsirivanit, Hisashi Narimatsu, Norie Araki

**Affiliations:** 1 Department of Tumor Genetics and Biology, Graduate School of Medical Sciences, Institute of Life Sciences, Kumamoto University, Kumamoto, Japan; 2 Research Center for Medical Glycoscience (RCMG), National Institute of Advanced Industrial Science and Technology (AIST), Tsukuba, Ibaraki, Japan; 3 Department of Biochemistry, Faculty of Medicine, Khon Kaen University, Khon Kaen, Thailand

**Keywords:** glioblastoma, cancer stem cells, proteomics, glycomics, chondroitin sulfate, differentiation

## Abstract

Glioma stem cell/glioma-initiating cell (GIC) and their niches are considered responsible for the therapeutic resistance and recurrence of malignant glioma. To clarify the molecular mechanisms of GIC maintenance/differentiation, we performed a unique integrated proteogenomics utilizing GIC clones established from patient tumors having the potential to develop glioblastoma. After the integration and extraction of the transcriptomics/proteomics data, we found that chondroitin sulfate proteoglycan 4 (CSPG4) and its glycobiosynthetic enzymes were significantly upregulated in GICs. Glyco-quantitative PCR array revealed that chondroitin sulfate (CS) biosynthetic enzymes, such as xylosyltransferase 1 (XYLT1) and carbohydrate sulfotransferase 11, were significantly downregulated during serum-induced GIC differentiation. Simultaneously, the CS modification on CSPG4 was characteristically decreased during the differentiation and also downregulated by XYLT1 knockdown. Notably, the CS degradation on CSPG4 by ChondroitinaseABC treatment dramatically induced GIC differentiation, which was significantly inhibited by the addition of CS. GIC growth and differentiation ability were significantly suppressed by CSPG4 knockdown, suggesting that CS–CSPG4 is an important factor in GIC maintenance/differentiation. To understand the molecular function of CS–CSPG4, we analyzed its associating proteins in GICs and found that CSPG4, but not CS–CSPG4, interacts with integrin αV during GIC differentiation. This event sequentially upregulates integrin–extracellular signal–regulated kinase signaling, which can be inhibited by cyclic-RGD (Arg-Gly-Asp) integrin αV inhibitor. These results indicate that CS–CSPG4 regulates the GIC microenvironment for GIC maintenance/differentiation *via* the CS moiety, which controls integrin signaling. This study demonstrates a novel function of CS on CSPG4 as a niche factor, so-called “glyco-niche” for GICs, and suggests that CS–CSPG4 could be a potential target for malignant glioma.

Malignant glioma is the most common and lethal primary brain tumor (1). Recently, glioma stem cells (GSCs)/glioma-initiating cells (GICs) have been proposed to be capable of extensive self-renewal, multilineage differentiation, and promotion of glioblastoma multiforme (GBM) development, and the most responsible for the therapeutic resistance and recurrence of GBM (2). It is suggested that GICs reside in a microenvironment referred to as the niche, which is composed of stem cells, neighboring supportive cells, extracellular matrix (ECM), and other factors required for stem cell maintenance or self-renewal. Hence, suppression or manipulation of GICs and their niches in terms of the GIC maintenance/self-renewal or differentiation to glioma cells may be the effective treatment strategy for malignant glioma. However, the molecular mechanisms or factors controlling GIC maintenance/differentiation have not been clearly identified.

To understand the mechanisms of GIC maintenance/differentiation, we previously established GIC clones having the potential to differentiate into malignant gliomas (3, 4) and analyzed the molecules upregulated in relation to GIC differentiation by our unique integrated proteogenomics (3). Using iPEACH (integrated Protein Expression Analysis CHart) (5), an original integration tool of transcriptome and proteome analysis data, we have shown for the first time that GICs specifically increase the secretion of ECM proteins, especially fibronectin (FN), and membrane expression of integrin αV (ITGAV) during the course of serum-induced differentiation. Importantly, the association of FN and ITGAV *via* the integrin motif, Arg-Gly-Asp (RGD), which represents a key factor in the GIC-mediated formation of a specific microenvironment, the so-called “differentiation niche,” was found to be necessary for early events in the differentiation and proliferation of GIC *in vitro* and *in vivo* (3). Interestingly, during the initial phase of GIC differentiation, treatment with an RGD peptide, which inhibits the integrin–FN interaction, significantly inhibited GIC proliferation as well as differentiation, and increased GIC sensitivity to the anticancer drug temozolomide. A combination treatment with temozolomide and RGD inhibited glioma progression and led to longer survival in a mouse intracranial GIC xenograft model, suggesting that the differentiation niche in the initial stage of the GIC could be a clinical target.

In this study, to clarify molecular mechanisms for the maintenance of GIC stemness and the switching system of GIC differentiation in detail, we tried to focus on the specific molecules highly expressed in the GIC spheres and downregulated during GIC differentiation. Our proteogenomics demonstrated that the expression levels of chondroitin sulfate proteoglycan 4 (CSPG4), especially the chondroitin sulfate (CS) modified form (CS–CSPG4), as well as cellular CS synthetic enzymes are significantly higher in the GIC spheres and downregulated during the differentiation. To understand the function of the CS on CSPG4 in GICs, we tried to decrease the CS modification on CSPG4 by treatment of the CS degradation enzyme or the knockdown of CS synthetic enzyme and found that the GICs dramatically start the differentiation after the CS degradation. We further analyzed CSPG4 function in relation to integrin signaling in the GIC differentiation and tried to demonstrate the possibility that CS-modified CSPG4 maintains GIC stemness by forming a specific microenvironment for GICs, the so-called “glyco-niche,” which inhibits the switching on the integrin-related differentiation/stimulation to the glioma development, and thus, CS, CSPG4, and the associating integrin signaling could be promising GIC therapy targets.

## Results

### Identification of glycoproteins in GICs using integrated proteogenomics

We previously established GIC clones from tumors of malignant glioma patients, having the potential to differentiate into malignant gliomas and form GBMs in mouse intracranial transplantation models (3, 4). These cells form spheres in the stem cell medium and have significant expression of neural stem cell (NSC) markers, such as SOX2 and CD133. Upon the stimulation by serum, which has been known as an inducer of GIC differentiation (6, 7, 8), the GIC spheres increase cellular proliferation, motility, and adhesion to the culture dishes and upregulate the glial cell markers such as GFAP (glial fibrillary acid protein) and the glioma marker CD44. Using these cells, our unique integrated proteogenomics were performed to survey the proteins and mRNAs differentially expressed in GICs after the differentiation (mRNA data are stored in Gene Expression Omnibus [GEO] with the accession number of GSE43762, proteome data are stored in jPOST (Japan ProteOme Standard Repository/database) with the accession numbers JPST0000355 and JPST0000361). To examine the molecules involved in GIC maintenance/differentiation, an iPEACH application (5), which can integrate information from several analysis types into a useful data file that provides comprehensive proteomics and transcriptomics data including functional annotations, was optimized and rearranged for this study. Upregulated and downregulated molecules in the differentiated GICs were listed with iPEACH score, which is the sum of the absolute value of the log fold change ratios obtained from DNA microarray and iTRAQ (8-Plex)-based proteomics data (all 11,594 molecules identified are shown in Table S1). The top 297 downregulated molecules during GIC differentiation (iPEACH score <−6) were subjected to Gene Ontology analysis. The most significant Gene Ontology groups were metabolic process (*p*: 5.70E-07), small-molecule metabolic process (*p*: 1.47E-08), small-molecule biosynthetic process (*p*: 6.47E-09), and so on (Table S2). In the small-molecule metabolic process group (78 molecules enriched), the first rank molecule was CSPG4 (iPEACH score = −16.545), and biosynthesis/transferase enzymes of glycosaminoglycans (GAGs) were also listed as the notable molecules (Table S3). CSPG4-associated enzymes, such as xylosyltransferase 1 (XYLT1: iPEACH score = −9.749) and carbohydrate sulfotransferase 11 (CHST11 iPEACH score = −6.291), were also significantly downregulated during GIC03A and GIC03U cell differentiation (Table S3). To further analyze the contribution of several glycosynthetic enzymes to GIC maintenance/differentiation, mRNA from GICs and serum-induced differentiated GIC cells were subjected to a quantitative real-time PCR array for 186 human glycogenes that encode proteins involved in glycan synthesis and modification (9). The results of glyco-quantitative PCR (qPCR) array showed that 58 of 186 glycogenes, including CS synthetic enzymes, such as XYLT1, CHST11, CHSY1, B3GAT3, CHST12, and B3GALT6, were downregulated in differentiated GICs (Table S4). These data strongly indicate that CSPG4 and related CS synthetic enzymes are highly associated with GIC maintenance/differentiation.

### CSPG4 was predominantly expressed in GIC spheres, and expression decreased during the differentiation process

**Figure 1:**
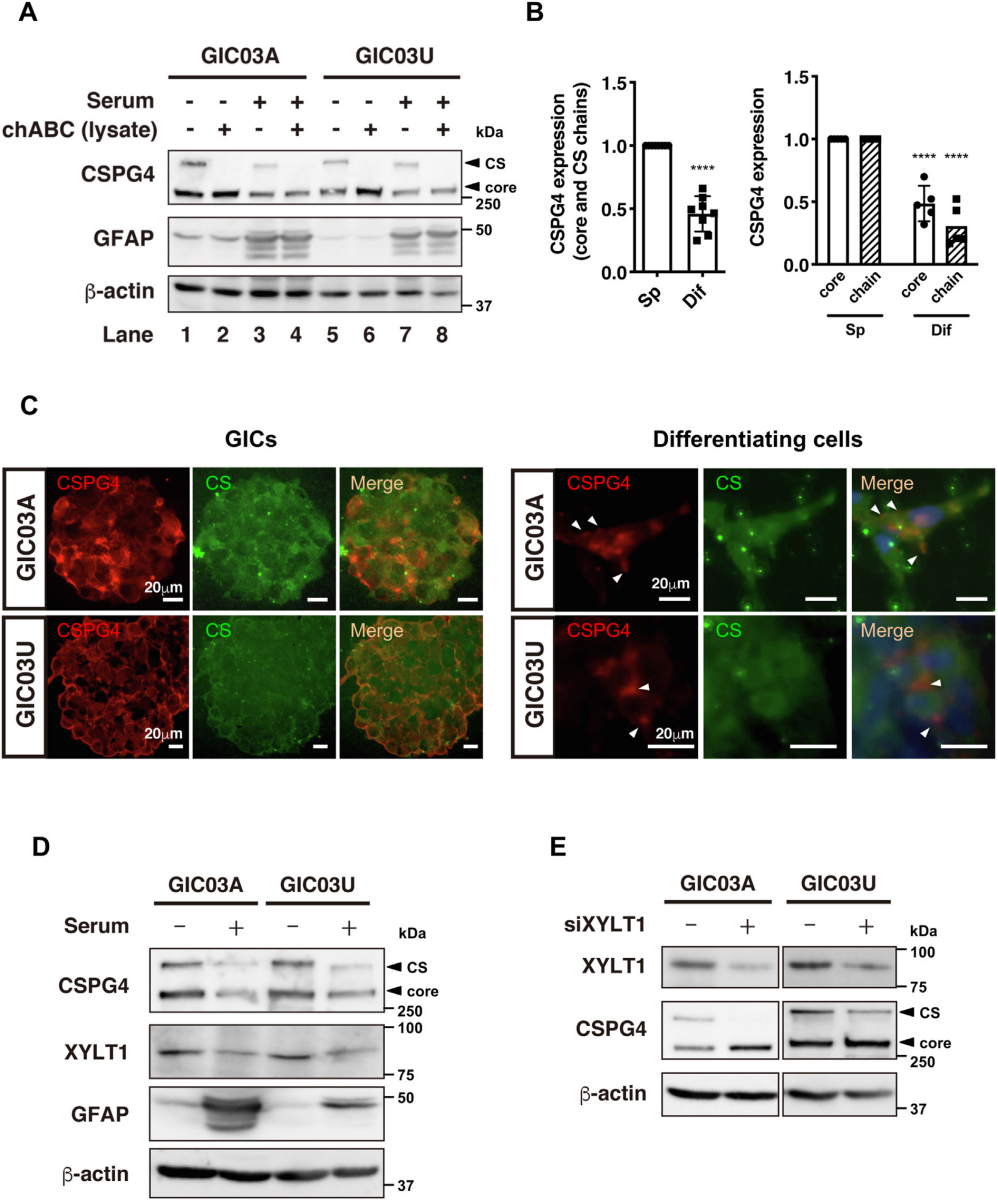
**CS-modified form of CSPG4 is predominantly expressed in GIC spheres, and its expression is correlated with XYLT1 expression, which is decreased in differentiating cells.***A*, validation of CSPG4 expression with and without serum stimulation and/or chABC treatment by Western blotting analysis. GICs or differentiating cells cultured in 10% serum for 5 days were lysed in RIPA buffer. The lysate was treated with 0.05 U chABC for 2 to 3 h at 37 °C and analyzed by Western blotting using antibodies against CSPG4 and GFAP. *Arrows* indicate CS-modified CSPG4 (*top*) and nonglycosylated core-type CSPG4 (*bottom*). GFAP was used for the differentiation marker of GICs to glioma cells. *B*, the intensity ratio of the total CSPG4 (core + CS modified) expression on GICs and differentiating cells (*left panel*) and the expression of nonglycosylated CSPG4 (CSPG4 core) or CS-modified CSPG4 (CS–CSPG4) on GICs or differentiating cells (*right panel*) analyzed in (*A*) was quantified using β-actin as an internal control. The values shown in (*B*) are the means ± SD of three independent experiments. Significance was tested with paired Student’s *t* test, ∗∗∗∗*p*< 0.0001. *C*, fluorescent immunocytochemistry for the analysis of CSPG4 and CS localization in GICs and serum-induced differentiating cells. GIC03A and GIC03U (*left*) or differentiating cells (*right*) after 5 days of culture were stained with anti-CSPG4 and anti-CS antibodies and visualized with Alexa Fluor 546 (*red*) or Alexa Fluor 488 (*green*)-conjugated secondary antibodies, respectively. The nuclei of differentiating cells were stained with 4′,6-diamidino-2-phenylindole (DAPI) (*blue*). *White arrowheads* indicate the cellular surface area stained with anti-CSPG4 antibodies. Scale bars represent 20 μm. *D*, Western blot analyses of CSPG4 and XYLT1 expressions in GICs and serum-induced differentiating cells. GFAP was used as a differentiation marker. GICs or differentiating cells by 10% serum for 5 days were lysed and followed by Western blot analysis. *E*, Western blotting analysis of the expression of XYLT1 and CSPG4 in GICs and serum-induced differentiating cells with siControl or siXYLT1. GICs were transfected with XYLT1 siRNA or control siRNA and cultured for 48 h. The analyses were performed using antibodies against XYLT1 and CSPG4. chABC, chondroitinaseABC; CS, chondroitin sulfate; GFAP, glial fibrillary acid protein; GIC, glioma-initiating cell; RIPA, radioimmunoprecipitation assay; XYLT1, xylosyltransferase 1.

To confirm the results obtained *via* integrated proteogenomics of GICs, the expression of CSPG4 was validated with Western blotting and immunocytochemical analyses. The Western blotting analysis using anti-CSPG4 antibodies showed that CSPG4 was predominantly expressed in GICs, and the expression decreased during differentiation of GIC03A and 03U cells (Fig. 1*A*: lanes 1, 3, 5, and 7; Fig. 1, *B* and *C*). Simultaneously, the astrocyte/glioma marker GFAP increased the expression upon serum stimulation (Fig. 1*A*: lanes 3, 4, 7, and 8). Interestingly, CSPG4 was identified as two protein bands with approximately 250 and 300 kDa on GIC spheres. CSPG4 is a type I transmembrane protein with a molecular weight of 250 kDa for its core protein or ∼300 kDa for the CS–GAG-modified form. To confirm the CSPG4 status in GICs, we treated the GIC cell lysates with chondroitinaseABC (chABC), a CS degradation enzyme, prior to Western blotting analysis. The high molecular weight 300 kDa band completely disappeared, and the lower 250 kDa band increased after digestion with chABC (Fig. 1*A*), suggesting that the 250 kDa band is nonglycosylated CSPG4 (CSPG4 core) and the 300 kDa band is CS–CSPG4. After serum stimulation, the expression level of CS–CSPG4 in GICs decreased significantly relative to that of the nonglycosylated form (Fig. 1*B*). Immunocytochemical analysis showed that CSPG4 and CS were apparently coexpressed on the surface of plasma membranes of GICs (Figs. 1*C*, *left panel* and S1*A*). To confirm the predominant cellular localization of CS on GICs, we treated the fixed GICs with chABC and determined that chABC clearly degraded and eliminated CS on CSPG4 in GICs (Fig. S1*A*), as similarly indicated in the Western blotting (Fig. 1*A*). Upon serum stimulation, CSPG4 and CS expressions were obviously downregulated in differentiating GICs. The immune staining of CS was found to be very faint and could not be assessed, whereas the CSPG4 protein expression was still observed on those differentiating GICs as shown in Figure 1*C*, *right panel* and Fig. S1, *A* and *B* (*arrows* indicate the CSPG4 staining on membrane area), suggesting that CS dissociation from CSPG4 could be related to GIC differentiation.

To clarify the CS segregation mechanism, we analyzed the expression of XYLT1, a catalytic enzyme for GAG assembly of CS and heparan sulfate (HS), using Western blot analysis. The results confirmed that both XYLT1 and CSPG4 were upregulated in GICs; in other words, XYLT1 and CS–CSPG4 were significantly downregulated during differentiation of GIC03A and 03U cells (Fig. 1*D*). To assess the effect of XYLT1 on GIC differentiation, GICs were transfected with XYLT1 siRNA. Treatment with XYLT1 siRNA significantly decreased CS–CSPG4 expression, in contrast to the cells treated with control siRNA (Fig. 1*E*). The increase of GFAP expression associated with the downregulation of CS–CSPG4 was reproducibly observed in the GICs after XYLT1 siRNA treatments (Fig. S2). These results suggest that GIC differentiation could be associated with inhibition of CS chain synthesis on CSPG4.

### CSPG4 CS glycochains suppress GIC differentiation

**Figure 2:**
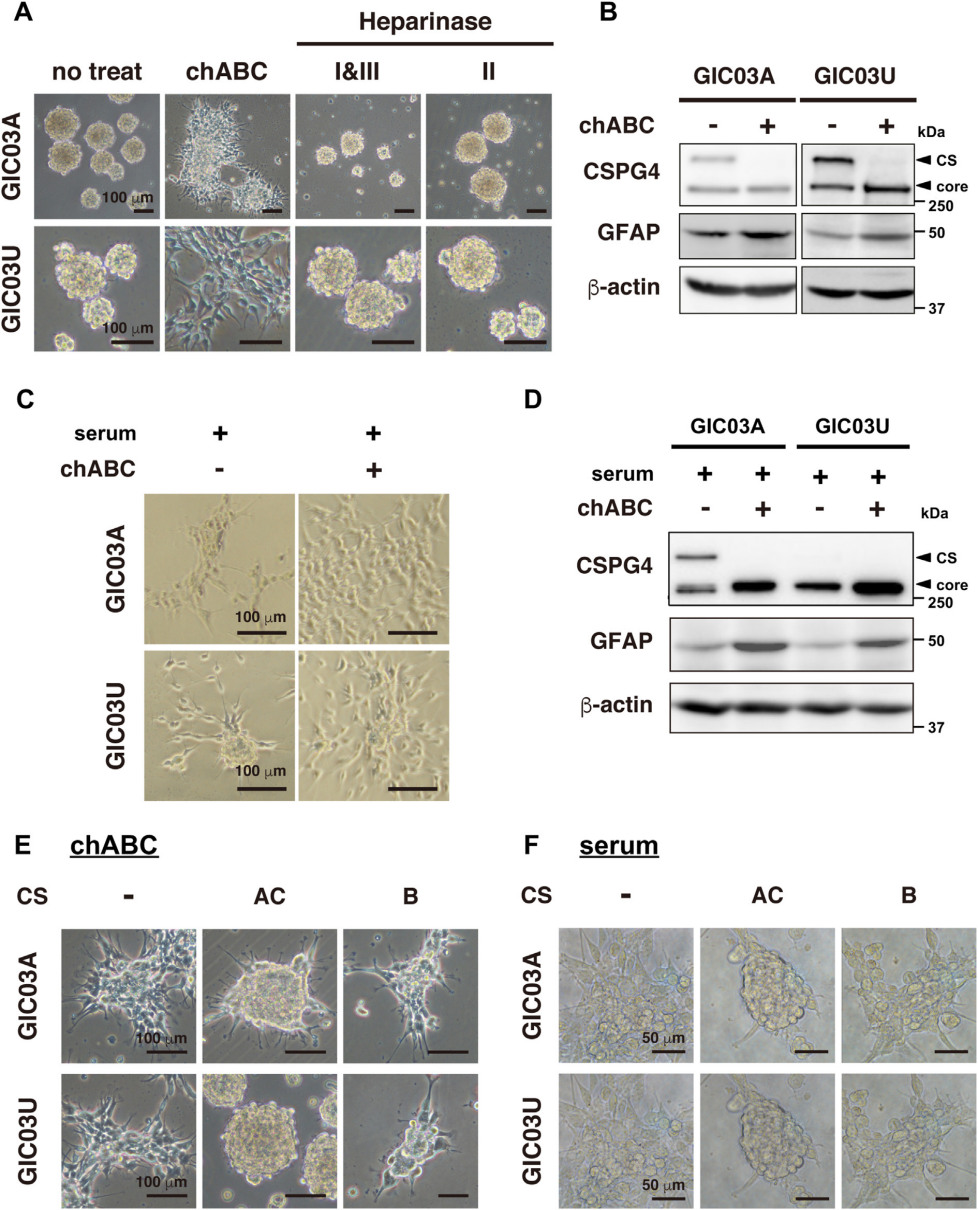
**CSPG4–chondroitin sulfate proteoglycan chains suppress GIC differentiation.** Cell morphology of GICs treated with chABC and heparinase I/III and II. GICs cultured in serum-free medium were treated with 0.05 U/ml of chABC, heparinase I/III, or heparinase II, followed by observation of cell morphology by microscopy after 48 h. Scale bars represent 100 μm. *B*, expression of CSPG4–CS and -core in GICs treated with chABC. GFAP was used as a differentiation marker. GICs were incubated with chABC for 48 h, followed by Western blot analysis. *Arrows* indicate CS-modified CSPG4 (*top*) and nonglycosylated core CSPG4 (*bottom*). *C*, morphology of serum-induced differentiating cells treated with or without chABC. GICs induced to differentiate in 1% serum were cultured with chABC, and morphological changes were observed after 24 h. Scale bars represent 100 μm. *D*, expression of CSPG4 and GFAP in serum-induced differentiating GICs with and without chABC. GICs were induced to differentiate in 1% serum and treated with or without chABC for 24 h. Cells were lysed and followed by Western blot analysis. *E* and *F*, the morphology of chABC- (*E*) or serum- (*F*) induced differentiating cells treated with 500 μg/ml of CS-AC or CS-B, respectively. Serum concentrations were used at 1% for GIC03A and 2% for GIC03U. Scale bars represent 100 μm (*E*) and 50 μm (*F*). chABC, chondroitinaseABC; CSPG4, chondroitin sulfate proteoglycan 4; GFAP, glial fibrillary acid protein; GIC, glioma-initiating cell.

To understand the role of CS–CSPG4 in GIC maintenance and differentiation, GIC spheres were treated with chABC directly in the GIC culture conditions, and the morphological changes were analyzed. Surprisingly, in serum-free NSC medium, the adhesion and migration of GIC spheres were dramatically increased within 4 h after chABC treatment (Figs. 2 and S3*A*), and the differentiation marker GFAP expression increased simultaneously (Fig. 2*B*) suggesting that chABC induces GIC differentiation. These phenotypic changes were not observed when spheres were treated with other glycolytic enzymes, such as heparinase I/III or heparinase II, which degrade heparin and HS on GAGs (Fig. 2*A*). Treatment with chABC under serum stimulation conditions also prominently stimulated cell migration and adhesion and GFAP expression (Figs. 2, *C* and *D*, and S3*B*).

To further study the function of CS in GIC differentiation, we next analyzed the effects of CS–GAGs, such as CS-A/C, the components of CS, and CS-B, the components of dermatan sulfate. Under stimulation with chABC, the differentiating cells were treated with CS-A/C or CS-B, and the cell morphological changes were analyzed. The results clearly showed that the dramatic enhancement of the adhesion/migration of GICs induced by chABC treatment was strongly inhibited by the addition of CS-A/C, whereas the effects of CS-B were minimal (Fig. 2*E*). In serum-induced differentiating cells, migration/adhesion was also suppressed by CS-A/C but not by CS-B (Fig. 2*F*). These results indicate that GIC differentiation is inhibited by the CS-glycochain of CS–CSPG4.

### CSPG4 siRNA suppresses GIC growth and differentiation

**Figure 3:**
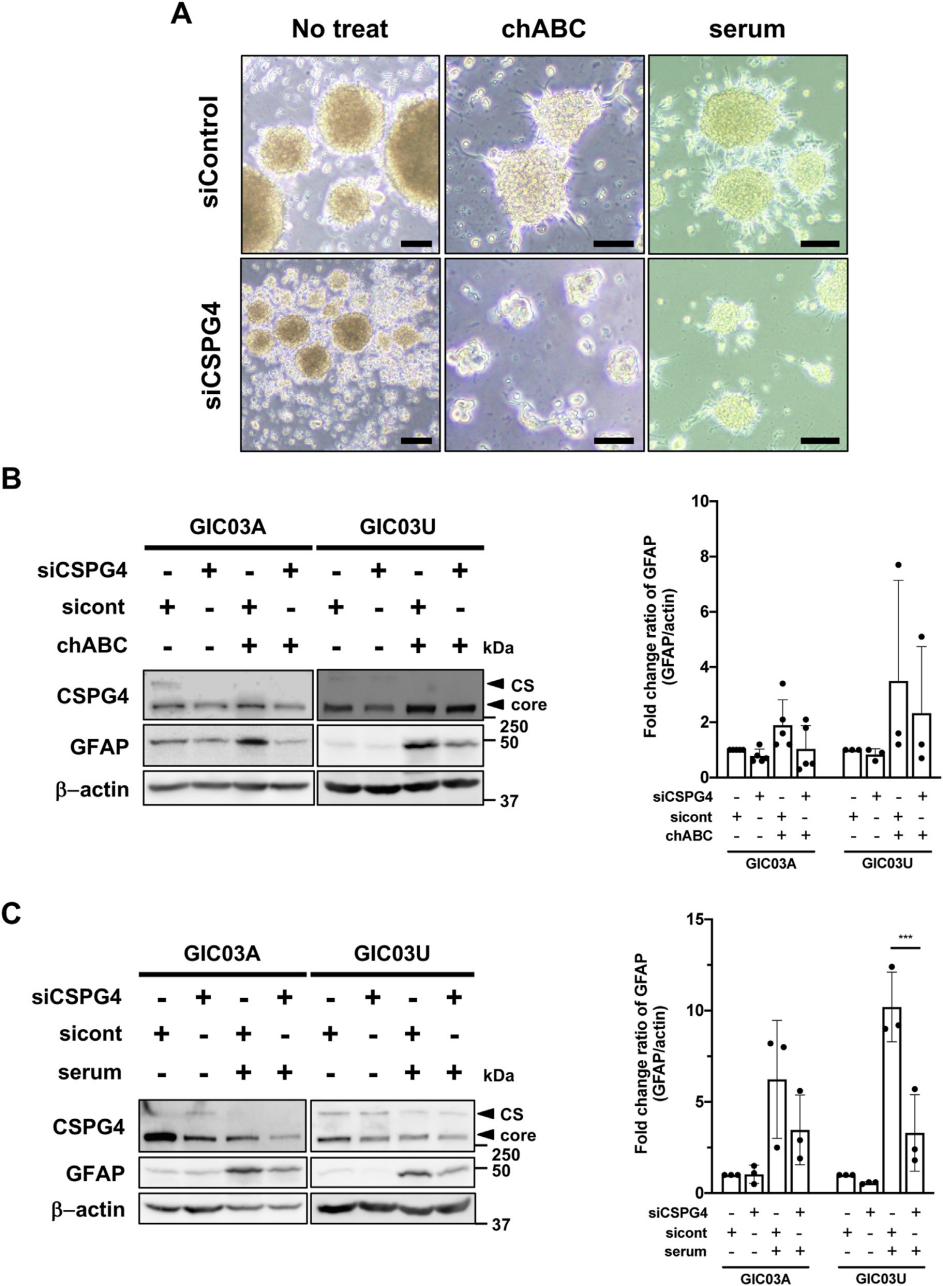
**CSPG4 regulates GIC differentiation.***A*, cell morphology of GICs transfected with siControl or siCSPG4 and induced to differentiate in chABC or serum. GICs were transfected with siControl or siCSPG4, cultured for 24 h, and then treated with chABC or 1% serum for 48 h. Scale bars represent 50 μm. *B* and *C*, effects of CSPG4 knockdown against GIC cell differentiation. After transfection with CSPG4 siRNA (siCSPG4) or control siRNA (sicont), GICs were cultured in serum-free medium for 24 h and then treated with chABC (*B*) or 1% serum (*C*) for 48 h. Cells were lysed and subjected to GFAP expression analysis. GFAP was quantified and normalized with β-actin, presented as the mean ± SD of three independent experiments. Significance was tested by multicomparison test. *C*, GIC03U: ∗∗∗*p* = 0.0004. ChABC, chondroitinaseABC; CSPG4, chondroitin sulfate proteoglycan 4; GFAP, glial fibrillary acid protein; GIC, glioma-initiating cell.

To examine the effect of CSPG4 deficiency on GIC differentiation, GICs were transfected with CSPG4 siRNA or control siRNA and cultured with chABC in NSC medium in the presence or the absence of serum, and cell phenotypes were analyzed. After 96 h of siRNA transfection, the migration/adhesion of GICs was greatly stimulated by control siRNA transfection with chABC or serum treatments, whereas these phenotypes were prominently suppressed by CSPG4 siRNA (Fig. 3*A*). In addition, compared with control siRNA, GIC spheres were morphologically damaged by CSPG4 siRNA, and GIC proliferation and sphere size were significantly decreased (Fig. S4, *A* and *B*). The expression of glial differentiation marker GFAP was upregulated up to 2- to 3.8-folds or 6- to 10-folds in both GICs upon chABC or serum treatments, respectively, and was reduced to less than half level or the level of nontreated GICs by CSPG4 knockdown (Fig. 3, *B* and *C*). These data suggest that CSPG4 is an important mediator in promoting GIC proliferation and differentiation.

### CSPG4–integrin interaction controls GIC maintenance/differentiation *via* extracellular signal–regulated kinase/mitogen-activated protein kinase (MAPK) signaling and can be inhibited by CS

**Figure 4:**
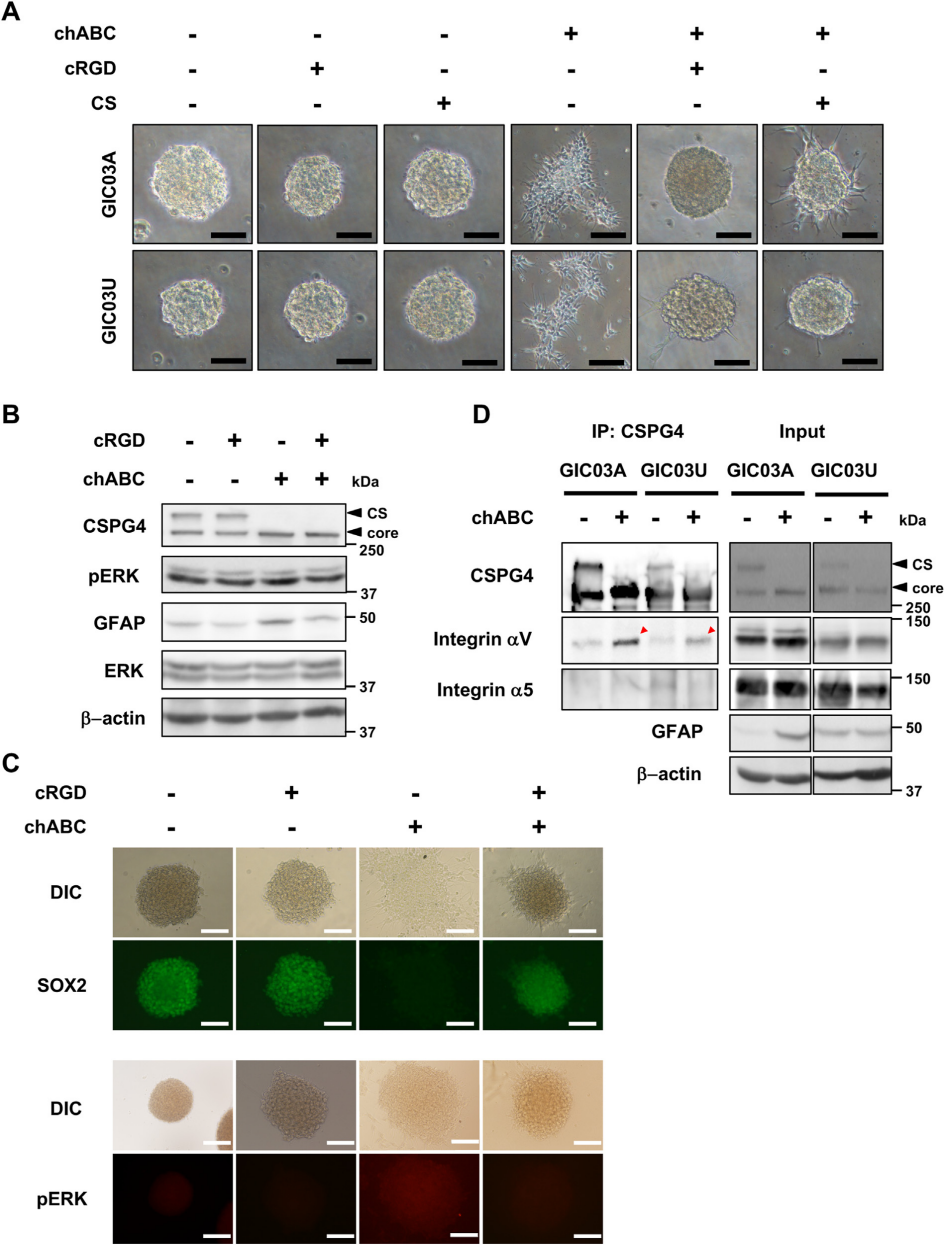
**CSPG4–integrin interaction induced by chABC upregulatesITGAV-dependent ERK/MAPK signaling.***A*, cell morphology of GICs and chABC-induced differentiating cells with or without cRGD or CS. Cells were cultured with 300 μM cRGD peptide or 500 μg/ml CS-A/C for 30 min followed by the treatment with or without chABC for 24 h. Scale bars represent 100 μm. *B*, phospho-ERK activation in GICs and chABC-induced differentiating cells. GICs were incubated with 500 μM cRGD peptide for 30 min and subsequently cultured with chABC for 48 h. pERK were analyzed by Western blot. *C*, effects of cRGD on SOX2 and phospo-ERK in GICs and chABC-induced differentiating cells. GICs and chABC-induced differentiating cells were cultured with or without cRGD peptide for 24 h. Fixed cells were stained with Alexa Fluor-488 (*green*) for SOX2 as a GIC marker and Alexa Fluor-546 (*red*) for pERK. Scale bars represent 100 μm. *D*, immunoprecipitation analysis to determine the binding between CSPG4 and ITGAV. Lysates from GICs or chABC-induced differentiating cells were subjected to immunoprecipitation using anti-CSPG4 antibody, followed by immunoblotting with anti-CSPG4, anti-ITGAV, or anti-integrin α5 antibodies (*left*). Whole-cell lysates (input) were analyzed by immunoblotting with antibodies against CSPG4, ITGAV, integrin α5, GFAP, and β-actin to assess each protein expression level (*right*). *Black arrowheads* showed CS-modified form of CSPG4 (CS) and a nonglycosylated core form of CSPG4 (core). *Red arrowheads* indicated the binding of ITGAV and CSPG4 in chABC-induced differentiating cells. ERK, extracellular signal–regulated kinase; chABC, chondroitinaseABC; cRGD, cyclic-Arg-Gly-Asp; CS, chondroitin sulfate; CSPG4, chondroitin sulfate proteoglycan 4; GIC, glioma-initiating cell; ITGAV, integrin αV; MAPK, mitogen-activated protein kinase; pERK, phospho-ERK.

Our previous report showed that the ECM and ITGAV *via* the RGD motif upregulated extracellular signal–regulated kinase (ERK)/MAPK signaling for GIC differentiation (3). Therefore, we speculated that the differentiation induced by chABC is also involved in integrin signaling (*via* the RGD motif). To test this possibility, GICs were cultured in chABC-containing medium in the presence of the integrin-specific binding peptide cyclic-Arg-Gly-Asp (cRGD) for 48 h, and GIC morphology and ERK/MAPK phosphorylation were analyzed. As expected, the induced migration/adhesion of cells after chABC treatment was significantly inhibited by cRGD (Fig. 4*A*). The Western blotting analyses and immunofluorescent staining of GICs showed that phosphorylated-ERK/MAPK upregulation and SOX2 downregulation were significant in the chABC-treated GICs (Figs. 4, *B* and *C*, and S5), whereas upon treatments with the cRGD peptide, those regulations were diminished or recovered, respectively (Figs. 4, *B* and *C*, and S5), indicating that the GIC differentiation and migration induced by chABC are mediated by integrin–ERK/MAPK signaling. These results demonstrate that segregation/degradation of the CS on CSPG4 in GICs upregulates GIC differentiation *via* upregulation of integrin–ERK/MAPK signaling.

Our previous report also showed that the ITGAV–FN interaction induces cell adhesion/migration and consequent GIC differentiation (3). Thus, it was also speculated that the integrin function induced in differentiation might be regulated by CSPG4. To confirm this possibility, we analyzed the interaction between integrins and CSPG4 on GICs by immunoprecipitation before and after induction of GIC differentiation with chABC. The GIC cell lysates were immunoprecipitated using anti-CSPG4 antibody, and the purified protein complexes were analyzed with anti-ITGAV antibody or anti-integrin α5 antibody as a control. Treatment with chABC significantly increased the interaction between CSPG4 and ITGAV but not with integrin α5. On the other hand, CS–CSPG4 before chABC treatment did not interact with either ITGAV or integrin α5 (Fig. 4*D*). These results suggest that CS–CSPG4 expressed in GICs inhibits the CSPG4 and ITGAV interaction, and CS degradation increased CSPG4–ITGAV binding and led to GIC differentiation.

### GBMs with high CSPG4 expression have correlations with CS modification enzymes and a poor prognosis of patients

**Figure 5:**
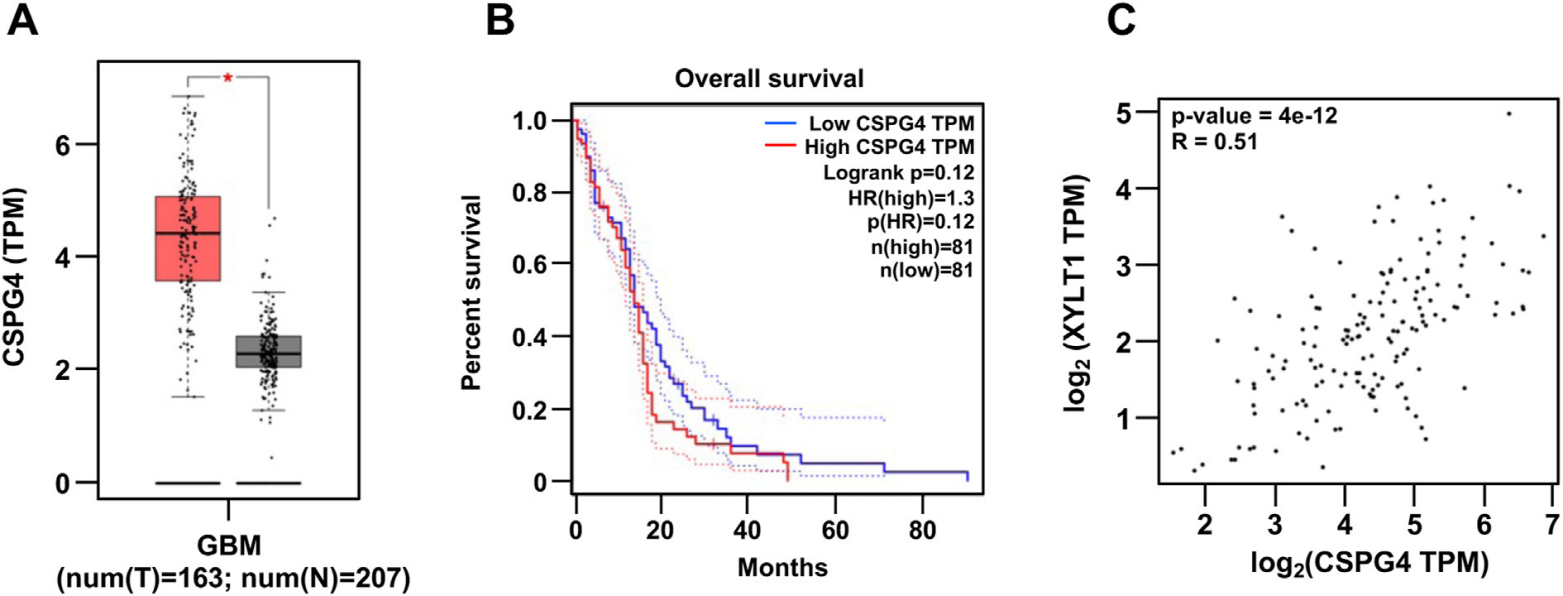
**GBMs with high CSPG4 expression have correlations with XYLT1 and a poor prognosis of patients.***A*, differential analysis of CSPG4 expression in tumors (num [T] = 163 samples) and normal samples (num [N] = 207 samples) using GBM transcriptome datasets in GEPIA. *B*, overall survival of GBM patients with high and low CSPG4 expression in GBM patients. *C*, correlation between CSPG4 and XYLT1 expressions in GBMs. CSPG4, chondroitin sulfate proteoglycan 4; GBM, glioblastoma multiforme; GEPIA, Gene Expression Profiling Interactive Analysis; XYLT1, xylosyltransferase 1.

To understand the clinical importance of CSPG4 and CS glycosylation in GBM patients, we further investigated the expression of CSPG4 and other CS-modified proteins (CSPG family proteins) in GBMs utilizing the GEPIA (Gene Expression Profiling Interactive Analysis) transcriptome public database. Among nine CS-modified proteins (CSPGs) reported in human, CSPG4 was significantly upregulated in GBMs compared with normal tissues (Fig. 5*A*) and was found to be associated with poor prognosis in GBM patients (Fig. 5*B*). We also found that there is a significant correlation between the expression of XYLT1 and CSPG4 in GBMs (Fig. 5*C*). Moreover, other chondroitin synthesis enzymes, such as CHST11, CHST12, and B3GALT6, were also highly correlated with the expression of CSPG4 in GBMs (Fig. S6).

CSPG4 and SMC3 among nine CSPGs were the only CS proteoglycans that decreased their expression during the induction of GIC differentiation; however, CSPG4 showed much higher expression level compared with SMC3 in GICs (Table S1 and Fig. S8). Although SMC3 was significantly expressed in GBMs compared with normal brain tissues (Fig. S7*A*), this expression did not affect patient prognosis (Fig. S7*B*). In addition, the correlation between XYLT1 and other eight CS proteins besides CSPG4 showed no correlation except SMC3 (Fig. S7, *C* and *D*). These results suggest that the CS modification on CSPG4 has an important role in GICs, which has a relation to GBM progression.

### CSPG4–integrin interaction induced by chABC upregulates ITGAV-dependent ERK/MAPK signaling

Based on our new findings in this study, we summarized the putative mechanisms of GIC maintenance and differentiation in the “glyco-niche” and “differentiation niche” and proposed the pathways, as shown in Fig. S9. In the GIC sphere conditions, CS–CSPG4 is expressed to form a “CSPG4-associated glyco-niche” for maintenance of GIC stemness. On the other hand, in the differentiation conditions, CS biosynthesis on CSPG4 is downregulated, and thus, the nonglycosylated CSPG4 is expressed on the cell surface. Subsequently, nonglycosylated CSPG4 binds to ITGAV with the ECM to form the “differentiation niche.” ERK/MAPK signaling is upregulated *via* CSPG4–ITGAV–ECM complexes and induces GIC differentiation to promote tumor formation. Manipulation of GIC glyco-niche degradation by chABC significantly induces GIC differentiation and upregulates ERK/MAPK signaling and subsequently causes tumorigenesis. Administration of cRGD or CS-A/B possibly inhibits the GIC differentiation and tumor formation.

## Discussion

In this study, to analyze the molecular mechanisms that control the maintenance of GIC stemness and induction of GIC differentiation, we performed an integrated proteogenomics using GIC clones established in our previous study (3) and identified molecules that are specifically upregulated in GICs and dynamically downregulated during the process of GIC differentiation into glioma cells.

We uniquely found that CSPG4 and related CS biosynthetic enzymes, such as XYLT1, CHST11, CHSY1, B3GAT3, CHST12, and B3GALT6, were upregulated in GIC and downregulated during serum-induced GIC differentiation (Fig 1, *A* and *B* and Table S4). Interestingly, during this differentiation process, CS modification of CSPG4 was markedly reduced. Treatment with a CS degradation enzyme (chABC) dramatically induced GIC differentiation without any other differentiation agent (Figs. 2 and S3). Knockdown of CSPG4 inhibited GIC proliferation and differentiation, suggesting that CS–CSPG4 regulates GIC maintenance/differentiation (Figs. 3 and S4). Moreover, CSPG4 interacted with ITGAV during GIC differentiation induced by CS segregation (Fig. 4). This event upregulated integrin–ERK/MAPK signaling, which was inhibited by the integrin inhibitor cRGD or the sugar-modified component of CSPG4, CS (Figs. 4 and S5). These results demonstrate for the first time that the glycan function of CS is important as a niche in cancer stem cells that supports GIC maintenance. It is postulated that this niche suppresses the integrin-associated differentiation signals, and that the suppression of CS niche function induces GIC differentiation.

Although multiple-omics has been expected to be a powerful method for understanding biological systems that can provide important information for clinical applications, integration of the results is challenging because of differences in the quantification algorithms used in each study (5). The iPEACH application can integrate multiple types of analytical information and provide comprehensive proteomics data including post-translational modifications, transcriptomics data, and functional annotations from several databases. In this analysis, using iPEACH scoring, CSPG4 and GAG chain biosynthesis enzymes were extracted as the most differentially expressed molecules in GIC (Table S1) and are expected to be candidate molecules to target. Thus, multiple-omics utilizing iPEACH could be useful for the clinical application.

CSPG4 is a type 1 membrane protein with a molecular weight of 250 kDa in its nonglycosylated (core) form and 300 kDa in the glycosylated (CS-modification) state (10). CSPG4 consists of a large extracellular domain with 2195 amino acids that makes up 96% of the protein, a transmembrane domain with 21 amino acids, and a short cytoplasmic tail of 77 amino acids (11, 12). Serine 995 in the extracellular domain has a characteristic CS modification. Originally, CSPG4 was identified in human melanoma cells (11) and neuroectoderm-derived tumors (13, 14) and has been suggested to be expressed in central nervous system cells during development/differentiation playing a critical role in proliferation and angiogenesis. Rodent CSPG4 has been known as “NG2” and often references either the protein itself or the highly proliferative/undifferentiated glial cells that express high levels of NG2 protein. NG2 glia represents the fourth major type of neuroglia in the mammalian nervous system and is classified as oligodendrocyte progenitor cells by virtue of their committed oligodendrocyte generation in the developing and adult brain (12). However, the molecular function of CSPG4 and CS on CSPG4 in NSCs or GICs, especially in human tissues or cells, has not been clearly identified.

CS–GAG-modified proteoglycans first begin with a consensus Ser-Gly/Ala-X-Gly motif, followed by the construction of a tetrasaccharide linker, where XYLT1/2, B4GALT7, B3GALT6, and B3GAT3 transfer. Addition of a GlcNAc moiety promotes the addition of GalNAc to the tetrasaccharide linker by the enzyme GalNAcT to initiate synthesis of CS–GAGs. The repetitive disaccharide region characteristic of CS is synthesized by the alternative addition of GlcA and GalNAc residues through the actions of GlcAT-II (GlcA transferase II, CHSY1/2, CHPF) and GalNAcT-I/II (GalNAc transferase I/II, CSGALNACT1/2), respectively, and these disaccharides are polymerized with chondroitin (sulfate) polymerizing factor (CHPF/CHSY1/CHSY3) (15). The 4-*O*-sulfation of GalNAc residues in CS is catalyzed by C4ST-1 (CHST11), C4ST-2 (CHST12), and C4ST-3 (CHST13) preferably (16). Our study using a glyco-qPCR array and integrated proteogenomics clearly showed that during GIC differentiation almost all these enzymes, such as XYLT1, B4GALT7, B3GAT3, C4ST-1 (CHST11), and C4ST-2 (CHST12), were downregulated (Table S4), strongly suggesting that CS–GAG synthesis on CSPG4 is important for GIC regulation.

Other known CS-modified proteins, such as SMC3, aggrecan, neurocan, versican, brevican, decorin, and biglycan, which are suggested to be expressed in glioma, melanoma, breast, pancreatic, and prostate cancer (17, 18, 19), were analyzed in detail with our study (Table S1 and Fig. S8) and found that only SMC3 was identified as an upregulated CSPG family protein in GICs besides the CSPG4. However, the expression level of SMC3 was much smaller than that of CSPG4 in GICs (Fig. S8). Moreover, the SMC3 expressions in GBM patients were not related to patient prognosis as opposed to those relation observed in CSPG4 *in silico* analysis (Figs. 5 and S8). Other CSPGs, except CSPG4 and SMC3, were not identified in GICs; instead, their increased expressions were observed in differentiating cells (Table S1), indicating that CSPG4 may be the most important CS carrier protein for the maintenance of GIC stemness.

In addition to CS, it is well known that HS and HS-modified proteoglycans (HSPGs), such as syndecan and glypican, are expressed in melanoma, breast cancer, glioma, and hepatocellular carcinoma and are related to cancer growth and metastasis (20, 21). In our study, the expression of these proteins was increased during GIC differentiation, suggesting that HSPGs could be important for GIC differentiation. However, in our analysis, when GIC spheres were treated with HS degradation enzymes, such as heparinase I/III or heparinase II, no phenotypic changes were observed in GICs (Fig. 2); whereas, the degradation of CS by chABC or inhibition of CS synthesis on CSPG4 protein by XYLT1 siRNA significantly induced GIC differentiation (Fig. 1). These results indicate that at least for maintenance of GIC stemness, the CS modification on CSPG4 is more important than the HS modification on HSPGs.

We speculate that CS–CSPG4 on the surface of the GICs could form a specific microenvironment, a so-called “stem niche,” a functional inhibitor of GIC differentiation signaling or a niche factor for the maintenance of GIC stemness. The niche is composed of soluble factors, ECM proteins, glycoproteins, and others that affect stem cell viability, proliferation, self-renewal, and fate determination. In the context of cancer stem/initiating cells, the niche also plays an important role in maintenance of stem cell function, tumor initiation, and protection against chemotherapeutic drugs. However, there have been no reports presenting evidence that CS or CS-modified proteins such as CSPG4 have obvious effects on GIC phenotypes. Our integrated proteogenomics data showed for the first time that CSPG4 might be one of the components of the GIC microenvironment or GIC-niche.

We previously reported that ITGAV interactions with the ECM produced by differentiating GICs are associated with GIC growth signaling and glioma progression *via* the formation of a specific microenvironment, the so-called “differentiation niche” (3). Here, we demonstrate that the GIC-niche factor CS of CSPG4 forms the stem cell niche, which can be broken by inhibition of CS synthesis and a decrease in CSPG4 expression *via* differentiation switching factors, creating a space for the interaction of CSPG4 protein with ITGAV and ECM to form the differentiation niche, followed by cellular induction of ERK/MAPK signaling (Fig. S9). CSPG4 has been suggested to interact with a number of proteins, such as type V and VI collagens in rat glial B49 and B111 cells (22), PDGFα receptor (23), multi-PDZ domain protein MUPP1 in CSPG4-overexpressing glioma U251 cells (24), galectin-3 (25), and syntenin-1 (26). Our immunoprecipitation experiment clearly showed that CSPG4 interacts with ITGAV in the GIC differentiation condition and stimulated GIC migration and differentiation. However, other CSPG4-interacting proteins may also contribute to the GIC differentiation niche, and we are currently studying this subject.

Numerous reports have implicated CSPG4 as a potential target for the treatment of malignant melanoma, breast cancer, and glioblastoma (27, 28, 29, 30). Anti-CSPG4 monoclonal antibody (28) and CSPG4 siRNA/shRNA (27, 29) inhibit melanoma breast cancer growth in mouse xenografts, indicating that lowering CSPG4 function/expression limits tumor growth and/or survival. It was also reported that CSPG4-targeted chimeric antigen receptor–redirected T cells efficiently controlled the growth of primary GBM cells (30). In this study, our results showed that CSPG4 knockdown suppressed cell proliferation, sphere formation, and differentiation of GICs (Fig. 3). Therefore, targeting CSPG4 could have direct effects on glioma stem cell maintenance/differentiation. Moreover, GIC differentiation increases the drug sensitivity (3), and thus, the strategy of the induction of the differentiation by the downregulation of the CS modification on CSPG4 may be utilized in the effective mobilization therapy of glioma. This study presents a novel function of CS and CSPG4 as a niche factor for GICs and suggests that CS–CSPG4 may be a novel clinical target for treating malignant glioma.

## Experimental procedures

### Reagents and antibodies

chABC, heparinase I/III, heparinase II, CS-A/C, and CS-B were purchased from Sigma–Aldrich. cRGD peptide was purchased from Peptide Institute, Inc. Anti-NG2 CS proteoglycan (AB5320) and anti-CS proteoglycan (MAB2029) antibodies were purchased from Millipore. Antibodies targeting NG2 (catalog no.: 4235), GFAP (catalog no.: 3670), p44/42 MAPK (Erk1/2) (catalog no.: 9102), and phospho-p44/42 MAPK (Erk1/2) (catalog no.: 9101) were purchased from Cell Signaling Technology. Anti-β-actin (catalog no.: 013-24553) was purchased from Wako Pure Chemical Industries. Antibodies targeting CS (ab11570) and ITGAV (ab179475) were purchased from Abcam. Antihuman integrin α5/CD49e antibody was purchased from R&D Systems. Anti-XYLT1 (catalog no.: 55061-1-AP) was purchased from Proteintech. Alexa Fluor 546-labeled goat anti-rabbit IgG, Alexa Fluor 546-labeled goat antimouse IgG, Alexa Fluor 488-labeled goat anti-rabbit IgG, and Alexa Fluor 488-labeled goat antimouse IgG were purchased from Invitrogen. Antimouse and anti-rabbit horseradish peroxidase–linked secondary antibodies were purchased from GE Healthcare. All siRNA oligonucleotides were purchased from Nippon Gene.

### Cell culture

The established GIC clones GIC03A, GIC03U, and GIC10025 were cultured in NSC medium: Neurobasal-A Medium (GIBCO/Invitrogen), B-27 (1:50 dilution; GIBCO/Invitrogen), heparin (5 μg/ml; SIGMA), and GlutaMax-1 (GIBCO) containing recombinant human fibroblast growth factor (20 ng/ml; PeproTech, Inc), recombinant human epidermal growth factor (20 ng/ml; PeproTech, Inc), and insulin (10 ng/ml; SIGMA). Each of the GIC clones was dissociated with Accumax (STEMCELL Technologies) and passaged once a week. All cells were used under the mycoplasma-free condition.

### Integrated proteome analysis

Transcriptomics and proteomics were performed as described previously (3). The proteomics raw data are available through the jPOST (31) (jPOST accession ID: JPST000361/PXD008332 and JPST000355/PXD008331). The DNA array raw data are available through the National Center for Biotechnology Information’s GEO (GEO series accession number: GSE43762). For the glycol-qPCR array, total RNA from GIC clones on day 2 of culture in NSC medium with or without 10% fetal calf serum was subjected to glycol-qPCR array analysis. Glyco-qPCR array data were combined with DNA microarray and proteomics data using the iPEACH (PCT/JP2011/58366).

### Western blot analysis

GIC lysates were dissolved in radioimmunoprecipitation assay (RIPA) buffer (50 mM Tris–HCl [pH 7.5]) containing 150 mM NaCl, 1% NP-40, 1% sodium deoxycholate, and protease and phosphatase inhibitor cocktails (Sigma) and subjected to SDS-PAGE. For CS proteoglycan digestion, cell lysates were incubated at 37 °C for 2 to 3 h and digested with 0.05 U of chABC prior to SDS-PAGE, followed by immunoblot analysis using anti-CSPG4 antibody. β-actin was used as the internal control. Enhanced chemiluminescence of horseradish peroxidase–conjugated antibodies was analyzed using an LAS4000 mini imaging system (GE Healthcare).

### Immunoprecipitation

For immunoprecipitation, cells were lysed with RIPA buffer containing 1% protease inhibitor cocktail. Cell lysates were incubated with anti-CS glycan antibody (clone: 9.2.27; Millipore) for 2 h, at 4 °C, followed by incubation with nProtein A Sepharose 4 Fast Flow (GE Healthcare) for 10 to 14 h, at 4 °C. Immunoprecipitates were washed three times with RIPA buffer and eluted in 2× Laemmli sample buffer that included β-mercaptoethanol. Samples were analyzed by immunoblotting.

### siRNA transfections

The following siRNA sequences were based on those used in a previous study: siRNA1 for human CSPG4 5′-GUGGACCAGUACCCUACGC-3′ and 5′-GUAGAUCAAUUGGGUACACUU-3′ as control siRNA (32). The following siRNA was designed: siRNA2 human CSPG4 5′-CCUUCCAUUAUGAGGUGGU-3’ (position 2549–2567) in human CSPG4 complementary DNA. siRNA for human XYLT1 was based on a previous study: 5′-GCAUCAUGCUACCAAUCUG-3’ (33). For RNA interference, cells were disassociated with Accumax, transfected with a CSPG4 siRNA1–siRNA2 mixture, XYLT1, or control siRNA using a Neon Transfection System (Thermo Fisher Scientific), Nucleofector (Lonza), or a NEPA21 Type II super electroporator (NEPAGENE Co), and cultured for 48 to 96 h. Cell morphological changes were photographed with an IX81 microscope (OLYMPUS).

### GIC differentiation assay

For GIC differentiation, GIC spheres were plated in NSC medium containing 1 to 10% serum, 0.05 U/ml chABC, and heparinase I/III or heparinase II for 48 h. In the inhibition experiments, GICs were pretreated with the integrin binding peptide cRGD or with the CS components, 500 μg/ml CS-A/C and CS-B, for 30 min before being seeded on a dish. Cellular morphological changes were photographed 48 h after treatment.

### Cell proliferation assay

After transfection with siRNA-CSPG4, GICs were seeded in 96-well plates (2 × 10^3^ cells/well). After transfection for 72 h, each well of cells was counted using Cell Counting Kit-8 (Dojindo) according to the manufacturer's recommendations, and cell surface area was measured with Metamorph software, version 7.5.5.0 using the area measurement mode (Molecular Device). Six replicates were examined for each condition.

### Immunocytochemical analysis

For CSPG4 and CS staining, cells were fixed with 4% paraformaldehyde, permeabilized, blocked with 5% bovine serum albumin, and reacted with primary antibodies: mouse monoclonal anti-CS proteoglycan (CSPG) antibody (Millipore), rabbit polyclonal anti-NG2 CS proteoglycan antibody (Millipore), and mouse monoclonal anti-CS antibody (Abcam) as described previously. For phospho-ERK1/2 and SOX2 staining, cells were fixed in 90% methanol for 30 min at room temperature. Fixed cells were blocked (5% bovine serum albumin, 1 h, room temperature) and reacted with the primary antibodies: rabbit polyclonal anti–phospho-p44/42 MAPK (Erk1/2) (Tht202/Tyr204) antibody and mouse monoclonal anti-SOX2 antibody (Cell Signaling Technology). Cells were washed with PBS and incubated with the appropriate secondary antibody: Alexa Fluor 488–conjugated goat antimouse, Alexa Fluor 546–conjugated goat anti-rabbit, or Alexa Fluor 546–conjugated goat antimouse antibody (Invitrogen). Nuclei were counterstained with 4′,6-diamidino-2-phenylindole, dilactate (Molecular Probes).

### *In silico* expression profiling

GEPIA (http://gepia.cancer-pku.cn/index.html) of the GBM transcriptome deposited in TCGA and GTEx datasets were used for obtaining the expression, correlation, and overall survival curves of CSPG family proteins, such as CSPG1: Aggrecan (ACAN), CSPG2: Versican (VCAN), CSPG3: Neurocan (NCAN), CSPG4: (melanoma-associated CS proteoglycan, NG2), CSPG5: Neuroglycan C (NGC), CSPG6: Bamacan (SMC3), CSPG7: Brevican (BCAN), and CSPG8: CD44, and Phosphacan, and CS glycan modification/synthetic enzymes such as XLTY1, CHST11, CHST12, and B3GALT6 in GBMs.

### Quantitation and statistical analysis

For quantitative Western blotting, the intensities of the chemiluminescence labels on each protein were visualized using an LAS4000 mini imager and quantified with ImageQuant TL software (GE Healthcare) using actin as an internal control. All the data are presented as the mean ± SD of three or more independent experiments run in duplicate. *p* Values were determined with Student’s *t* test or Tukey’s test using GraphPad Prism, version 7.01 (GraphPad Software, Inc). ∗*p* < 0.05, ∗∗*p* < 0.01, ∗∗∗*p* < 0.001, and ∗∗∗∗*p* < 0.0001 were considered significant.

## Data availability

The data supporting this study's findings are available from the corresponding author upon reasonable request.

## Supporting information

This article contains supporting information.

## Conflict of interest

The authors declare that they have no conflicts of interest with the contents of this article.

## References

[1] Stupp R., Pavlidis N., Jelic S., Force E.G.T. (2005). ESMO Minimum Clinical Recommendations for diagnosis, treatment and follow-up of malignant glioma. Ann. Oncol..

[2] Pollard S.M., Yoshikawa K., Clarke I.D., Danovi D., Stricker S., Russell R. (2009). Glioma stem cell lines expanded in adherent culture have tumor-specific phenotypes and are suitable for chemical and genetic screens. Cell Stem Cell.

[3] Niibori-Nambu A., Midorikawa U., Mizuguchi S., Hide T., Nagai M., Komohara Y. (2013). Glioma initiating cells form a differentiation niche via the induction of extracellular matrices and integrin alphaV. PLoS One.

[4] Putthisen S., Silsirivanit A., Panawan O., Niibori-Nambu A., Nishiyama-Ikeda Y., Ma-In P. (2022). Targeting alpha2,3-sialylated glycan in glioma stem-like cells by Maackia amurensis lectin-II: a promising strategy for glioma treatment. Exp. Cell Res..

[5] Hirayama M., Kobayashi D., Mizuguchi S., Morikawa T., Nagayama M., Midorikawa U. (2013). Integrated proteomics identified novel activation of dynein IC2-GR-COX-1 signaling in neurofibromatosis type I (NF1) disease model cells. Mol. Cell Proteomics.

[6] Yoo Y.D., Lee D.H., Cha-Molstad H., Kim H., Mun S.R., Ji C. (2017). Glioma-derived cancer stem cells are hypersensitive to proteasomal inhibition. EMBO Rep..

[7] Gu D., Zhou F., You H., Gao J., Kang T., Dixit D. (2023). Sterol regulatory element-binding protein 2 maintains glioblastoma stem cells by keeping the balance between cholesterol biosynthesis and uptake. Neuro Oncol..

[8] Haas T.L., Sciuto M.R., Brunetto L., Valvo C., Signore M., Fiori M.E. (2017). Integrin α7 is a functional marker and potential therapeutic target in glioblastoma. Cell Stem Cell.

[9] Narimatsu H., Sawaki H., Kuno A., Kaji H., Ito H., Ikehara Y. (2010). A strategy for discovery of cancer glyco-biomarkers in serum using newly developed technologies for glycoproteomics. FEBS J..

[10] Price M.A., Colvin Wanshura L.E., Yang J., Carlson J., Xiang B., Li G. (2011). CSPG4, a potential therapeutic target, facilitates malignant progression of melanoma. Pigment Cell Melanoma Res.

[11] Wilson B.S., Imai K., Natali P.G., Ferrone S. (1981). Distribution and molecular characterization of a cell-surface and a cytoplasmic antigen detectable in human melanoma cells with monoclonal antibodies. Int. J. Cancer.

[12] Yadavilli S., Hwang E.I., Packer R.J., Nazarian J. (2016). The role of NG2 proteoglycan in glioma. Transl. Oncol..

[13] Stallcup W.B., Huang F.J. (2008). A role for the NG2 proteoglycan in glioma progression. Cell Adh. Migr..

[14] Rettig W.J., Real F.X., Spengler B.A., Biedler J.L., Old L.J. (1986). Human melanoma proteoglycan: expression in hybrids controlled by intrinsic and extrinsic signals. Science.

[15] Mikami T., Kitagawa H. (2013). Biosynthesis and function of chondroitin sulfate. Biochim. Biophys. Acta.

[16] Kluppel M. (2010). The roles of chondroitin-4-sulfotransferase-1 in development and disease. Prog. Mol. Biol. Transl. Sci..

[17] Edwards I.J. (2012). Proteoglycans in prostate cancer. Nat. Rev. Urol..

[18] Barkovskaya A., Buffone A., Zidek M., Weaver V.M. (2020). Proteoglycans as mediators of cancer tissue mechanics. Front. Cell Dev. Biol..

[19] Theocharis A.D., Tsolakis I., Tzanakakis G.N., Karamanos N.K. (2006). Chondroitin sulfate as a key molecule in the development of atherosclerosis and cancer progression. Adv. Pharmacol..

[20] Szatmari T., Otvos R., Hjerpe A., Dobra K. (2015). Syndecan-1 in cancer: implications for cell signaling, differentiation, and prognostication. Dis. Markers.

[21] Sung Y.K., Hwang S.Y., Park M.K., Farooq M., Han I.S., Bae H.I. (2003). Glypican-3 is overexpressed in human hepatocellular carcinoma. Cancer Sci..

[22] Tillet E., Ruggiero F., Nishiyama A., Stallcup W.B. (1997). The membrane-spanning proteoglycan NG2 binds to collagens V and VI through the central nonglobular domain of its core protein. J. Biol. Chem..

[23] Tian X.Y., Zhang L., Sun L.G., Li M. (2015). Epigenetic regulation of miR-129-2 leads to overexpression of PDGFRa and FoxP1 in glioma cells. Asian Pac. J. Cancer Prev..

[24] Barritt D.S., Pearn M.T., Zisch A.H., Lee S.S., Javier R.T., Pasquale E.B. (2000). The multi-PDZ domain protein MUPP1 is a cytoplasmic ligand for the membrane-spanning proteoglycan NG2. J. Cell Biochem..

[25] Fukushi J., Makagiansar I.T., Stallcup W.B. (2004). NG2 proteoglycan promotes endothelial cell motility and angiogenesis via engagement of galectin-3 and alpha3beta1 integrin. Mol. Biol. Cell.

[26] Chatterjee N., Stegmuller J., Schatzle P., Karram K., Koroll M., Werner H.B. (2008). Interaction of syntenin-1 and the NG2 proteoglycan in migratory oligodendrocyte precursor cells. J. Biol. Chem..

[27] Wang J., Svendsen A., Kmiecik J., Immervoll H., Skaftnesmo K.O., Planaguma J. (2011). Targeting the NG2/CSPG4 proteoglycan retards tumour growth and angiogenesis in preclinical models of GBM and melanoma. PLoS One.

[28] Wang X., Osada T., Wang Y., Yu L., Sakakura K., Katayama A. (2010). CSPG4 protein as a new target for the antibody-based immunotherapy of triple-negative breast cancer. J. Natl. Cancer Inst..

[29] Yu L., Favoino E., Wang Y., Ma Y., Deng X., Wang X. (2011). The CSPG4-specific monoclonal antibody enhances and prolongs the effects of the BRAF inhibitor in melanoma cells. Immunol. Res..

[30] Pellegatta S., Savoldo B., Di Ianni N., Corbetta C., Chen Y., Patane M. (2018). Constitutive and TNFalpha-inducible expression of chondroitin sulfate proteoglycan 4 in glioblastoma and neurospheres: implications for CAR-T cell therapy. Sci. Transl. Med..

[31] Okuda S., Watanabe Y., Moriya Y., Kawano S., Yamamoto T., Matsumoto M. (2017). jPOSTrepo: an international standard data repository for proteomes. Nucleic Acids Res..

[32] Joo N.E., Watanabe T., Chen C., Chekenya M., Stallcup W.B., Kapila Y.L. (2008). NG2, a novel proapoptotic receptor, opposes integrin alpha4 to mediate anoikis through PKCalpha-dependent suppression of FAK phosphorylation. Cell Death Differ..

[33] Dziedzic D., Wegrzyn G., Jakobkiewicz-Banecka J. (2010). Impairment of glycosaminoglycan synthesis in mucopolysaccharidosis type IIIA cells by using siRNA: a potential therapeutic approach for Sanfilippo disease. Eur. J. Hum. Genet..

