# Supplementary material for: Chondroitin sulfate modification of CSPG4 regulates the maintenance and differentiation of glioma-initiating cells via integrin-associated signaling
Source: J Biol Chem. 2024 Feb 2;300(3):105706. doi: 10.1016/j.jbc.2024.105706 (PMC10958118; doi:10.1016/j.jbc.2024.105706)
Supplement: Supporting information [file mmc1.pdf]

Chondroitin sulfate modification of CSPG4 regulates the maintenance and differentiation of glioma initiating cells via integrin-associated signaling

Running title: *The role of chondroitin sulfate in the glioma stem cell*

Akiko Niibori-Nambu[1], Yoshimune Yamasaki[1], Daiki Kobayashi[1], Kiyohiko Angata[2], Atsushi Kuno[2], Orasa Panawan[1,3], Atit Silsirivanit[1,3], Hisashi Narimatsu[2] and Norie Araki[1*]

[1]Department of Tumor Genetics and Biology, Graduate School of Medical Sciences, Institute of Life Sciences, Kumamoto University, Kumamoto 860-8556, Japan

[2]Research Center for Medical Glycoscience (RCMG), National Institute of Advanced Industrial Science and Technology (AIST), Tsukuba, Ibaraki, 305-8568, Japan

[3]Department of Biochemistry, Faculty of Medicine, Khon Kaen University, Khon Kaen, 40002, Thailand

Corresponding author information: Norie Araki: Ph.D., Department of Tumor Genetics and Biology, Graduate School of Medical Sciences, Institute of Life Sciences, Kumamoto University, 1-1-1, Honjo, Chuo-ku, Kumamoto 860-8556, Japan; nori@gpo.kumamoto-u.ac.jp;
Tel: +81-96-373-5119; Fax: +81-96-373-5210

List of supporting information

Table S1 - S4 Legends, Detailed data are in the separated Excel files (Table S1-S4).
Figure S1 – S7

Supplemental Table S1. List of the identified molecules and associated data obtained by DNA microarray and iTRAQ with iPEACH score (Detailed data is in the separated Excel file as Table S1). Quantification of proteins was carried out based on the relative intensities of iTRAQ reporter ion released during MS/MS fragmentation of peptides according to the Paragon algorithm of ProteinPilot Version 4.5. iTRAQ indices were calculated by the sum of fold change ratio obtained from iTRAQ (MALDI/Elite/5600) data. Every annotation with merged data was obtained using iPEACH software.

UniProt release 2011_01 was used for the annotation.

*iPEACH indices (z) were calculated as follows:

$z = \Sigma\{log2\ (03A dif2/03A sp2) + log2\ (03A dif7/03A sp7) + log2\ (03U dif2/03U sp2) + log2\ (03U dif7/03U sp7)\}$

03A*sp2*, 03A*sp7*, 03A*dif2*, 03A*dif7*, 03U*sp2*, 03U*sp7*, 03U*dif2* and 03U*dif7* = ratio of gene or protein expression in the differentiation condition to sphere condition after 2 and 7 days of cell culture.

Each suffix indicates culture periods for the differentiation or sphere condition.

Column legends were shown in the table below:

Entrez_ID	Entrez_ID
UniProt_ID	UniProt_ID
Review	The data source from SwissProt or trEMBL
UniProt_Accession	UniProt_Accession
Gene_Name	Gene_Name from UniProt
Name	Name
Method/Master_Spot Number	Method/Master_Spot_Number
GIC03A sp7/sp2	Ratio Obtained from DNA microarray, iTRAQ (ESI/MALDI) data at each day point denominatered by sp2 ratio.
GIC03A dif2/sp2	
GIC03A dif7/sp2	
GIC03U sp7/sp2	
GIC03U dif2/sp2	
GIC03U dif7/sp2	
log2_03A_dif2/sp2	Fold changes (values as dif2/sp2 and dif7/sp7 calculated from each day point denominatered by sp2 ratio of 03A and 03U, and computed the log (base 2)) obtained from DNA microarray, iTRAQ (ESI/MALDI) data
log2_03A_dif7/sp7	
log2_03U_dif2/sp2	
log2_03U_dif7/sp7	
Absolute_log2_03A_dif2/sp2	Fold changes (absolute value of each above-mentioned log raio of 03A and 03U) obtained from DNA microarray, 2D-DIGE, iTRAQ (ESI/MALDI) data at each time point dominated by sp2 ratio.
Absolute_log2_03A_dif7/sp7	
Absolute_log2_03U_dif2/sp2	
Absolute_log2_03U_dif7/sp7	
log2_dif2/sp2_dif7/sp7	Each stated of log2 (dif2/sp2) + log2 (dif7/sp7) ratio obtained from DNA microarray, iTRAQ (ESI/MALDI) data and those absolute values.
Absolute_log2_dif2/sp2_dif7/sp7	
iPEACH_log2_dif2/sp2_dif7/sp7	iPEACH score 1, the sum fold changes (log2 (dif2/sp2) + log2 (dif7/sp7)) obtained from DNA microarray and iTRAQ (ESI/MALDI) data.
iPEACH_Absolute_log2_dif2/sp2_dif7/sp7	
Modifications	Post Translational Modifications identified by iTRAQ
Cleavages	Protein cleaves sites identified by iTRAQ
GeneOntology Biological Process	Related terms of Gene Ontology Annotation (Biological Process)
Gene Ontolology Cellular Component	Related terms of Gene Ontology Annotation (Molecular Function)
Gene Ontology Molecular Function	Related terms of Gene Ontology Annotation (Cellular Components)

Supplemental Table S2.

Overrepresented biological processes determined by GO enrichment analysis of mRNA and proteins differentially expressed in response to serum stimulation.

297 molecules with an iPEACH score less than -6 were used for further GO enrichment analysis. The table shows the highly extracted GO terms with biological process grouped as downregulated molecules during GIC differentiation. The table is reflected in the graph.

Supplemental Table S3.

List of "small molecule metabolic process" identified by “Biological Process of GO analysis". The molecules were extracted from the “small molecules metabolic process” GO term.

Supplemental Table S4.

List of genes identified by glyco-qPCR array.

Glyco-qPCR array ratios were obtained from the ratio of the mRNA expression in the differentiation condition to GIC condition after 2 days of cell culture. Average ratio is the sum of fold changes ratio of GIC03A and GIC03U.

Figure S1

A

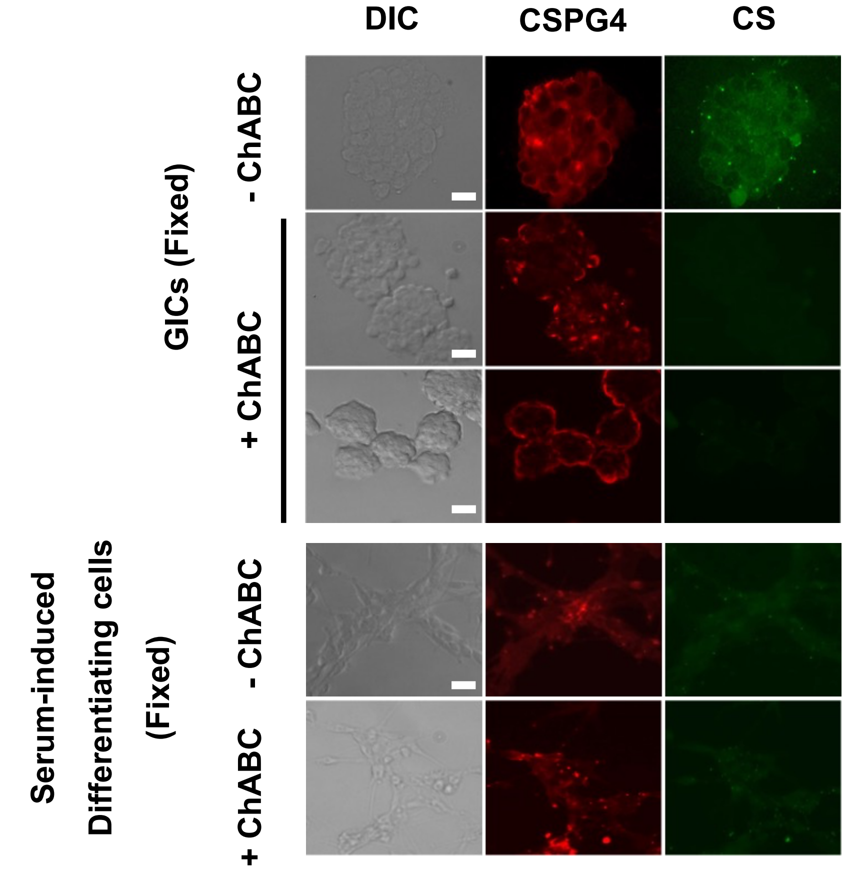
DIC
CSPG4
CS
GICs (Fixed)
- ChABC
+ ChABC
Serum-induced
Differentiating cells
(Fixed)
- ChABC
+ ChABC

B

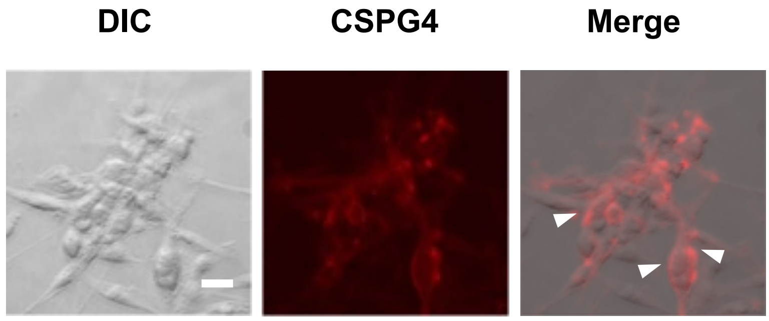
DIC
CSPG4
Merge

Figure S1. Immunocytochemistry of the CSPG4 and CS in GICs, with or without chABC treatments, and serum and chABC induced differentiating cells. (A) GIC spheres were treated with or without 0.05 U/ml of chABC at 37 °C for 2 hours after fixation with 4% paraformaldehyde. GIC differentiating cells induced by serum stimulation for 72h were also treated with or without 0.05 U/ml of chABC at 37 °C for 2 hours after fixation with 4% paraformaldehyde. Fixed cells were stained with anti-CSPG4 (AlexaFlour 488, green) or anti-CS antibody (AlexaFlour 546, red). Scale bars, 50 μm. (**B**) Differentiating cells induced by 10% serum, fixed with 4% paraformaldehyde and stained with anti-CSPG4 antibody (AlexaFlour 546, red) are shown. Arrows show the cellular surface staining of CSPG4. Scale bars, 25 μm.

Figure S2

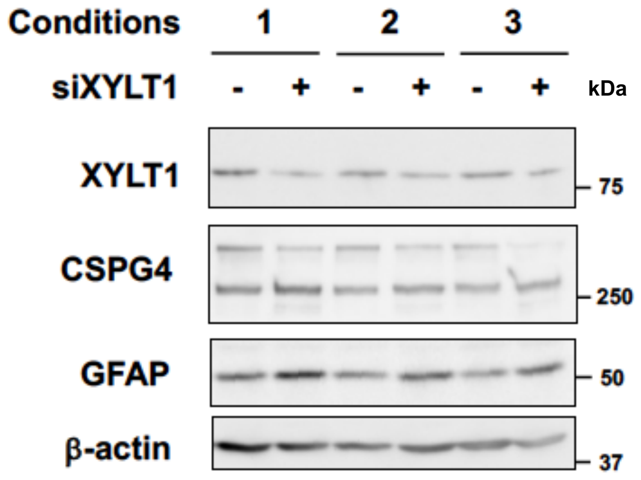

Figure S2. Western Blotting of GICs treated with siXYLT1 to analyze CSPG4 and GFAP expressions. GICs were treated with siXYLT and siControl under several transfection conditions by electroporation (Condition 1: 1200 mA, 20 v, 2ms; Condition 2: 1400 mA, 20 v, 2ms; Condition 3: 1350 mA, 30 v, 2ms), cultured for 72 h, and analyzed by the western blotting using anti-CSPG4, GFAP (a differentiation marker), and β-actin antibodies (an internal control).

Figure S3

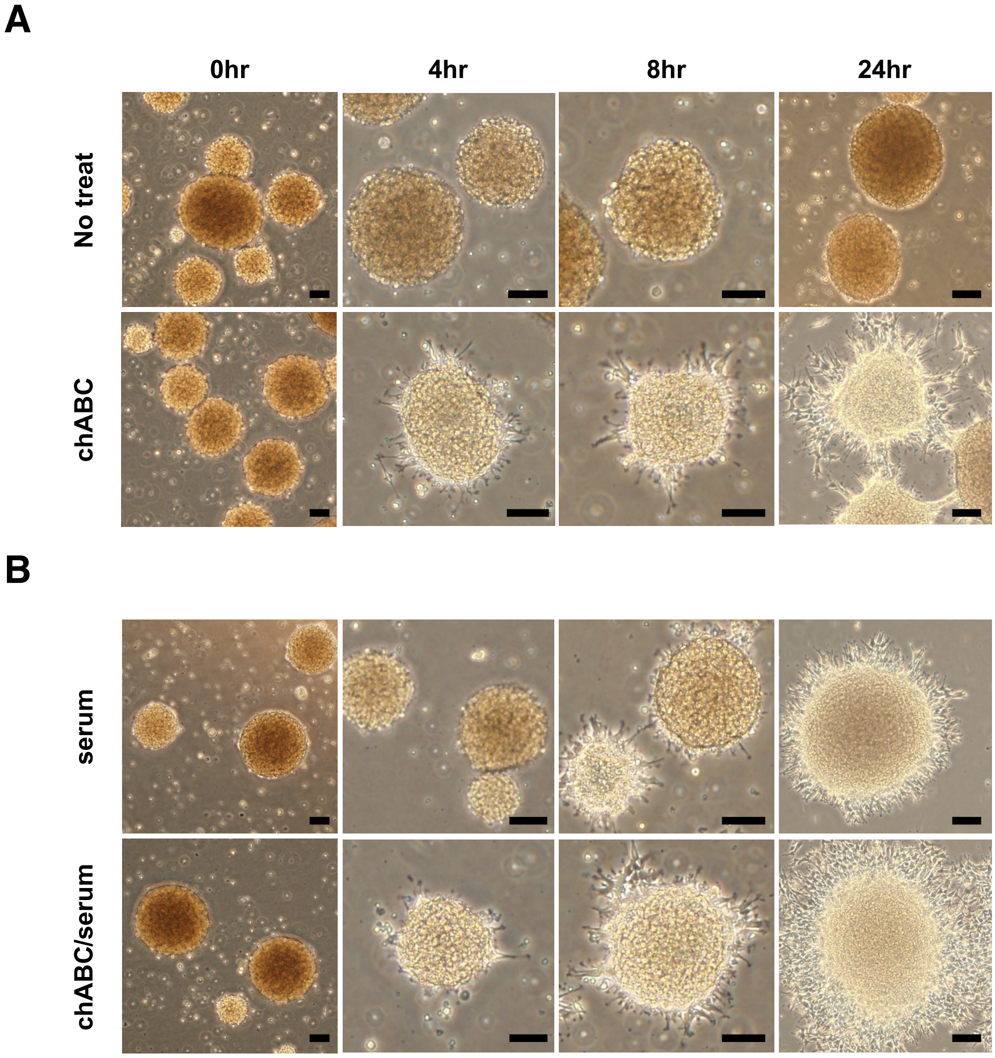

Figure S3. Phase contrast microscopic observations of the GIC differentiation induced by chABC and serum treatments. (A) GIC spheres (representative GICs in each group before treatments are presented as “at 0 hr”) treated with 0.05 U/ml l of chABC in NSC medium, and (**B**) 1% FCS (serum) or both of chABC and serum (0.05 U/ml and 1%, respectively), for 4, 8, and 24 h of their stimulations are shown. Scale bars, 100 μm.

Figure S4

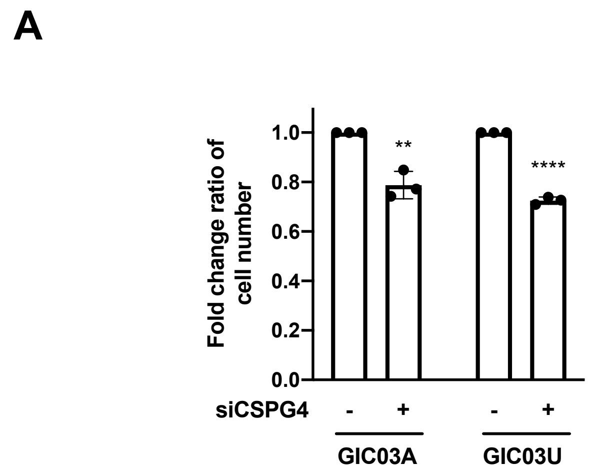

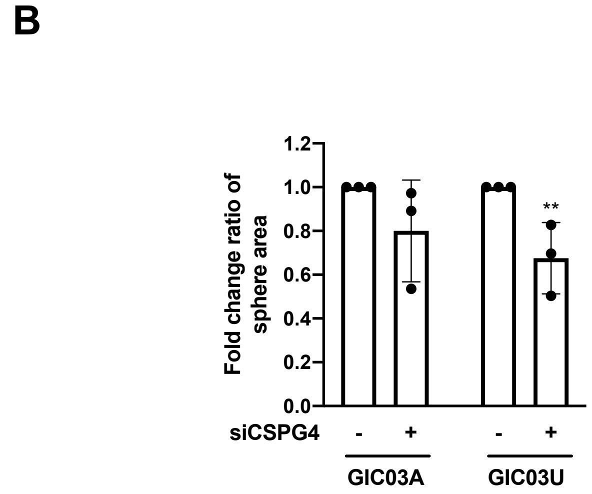

Figure S4. CSPG4 siRNA downregulates GIC proliferation. (A) GICs were transfected with CSPG4 siRNA or control siRNA for 72 h in NSC medium, and cell proliferation was analyzed with CCK-8 assay. **(B)** GICs were transfected with CSPG4 siRNA or control siRNA for 72 h, and the sphere areas were measured using Metamorph software (Molecular Devices). The fold change ratio values shown in (A) and (B) are the means ±S.D. of three or more independent experiments. Significance was tested with a paired Student's *t*-test. (A) GIC03A: $p = 0.0026$, GIC03U: ****$p < 0.0001$, (B) GIC03U: $p = 0.0097$

Figure S5

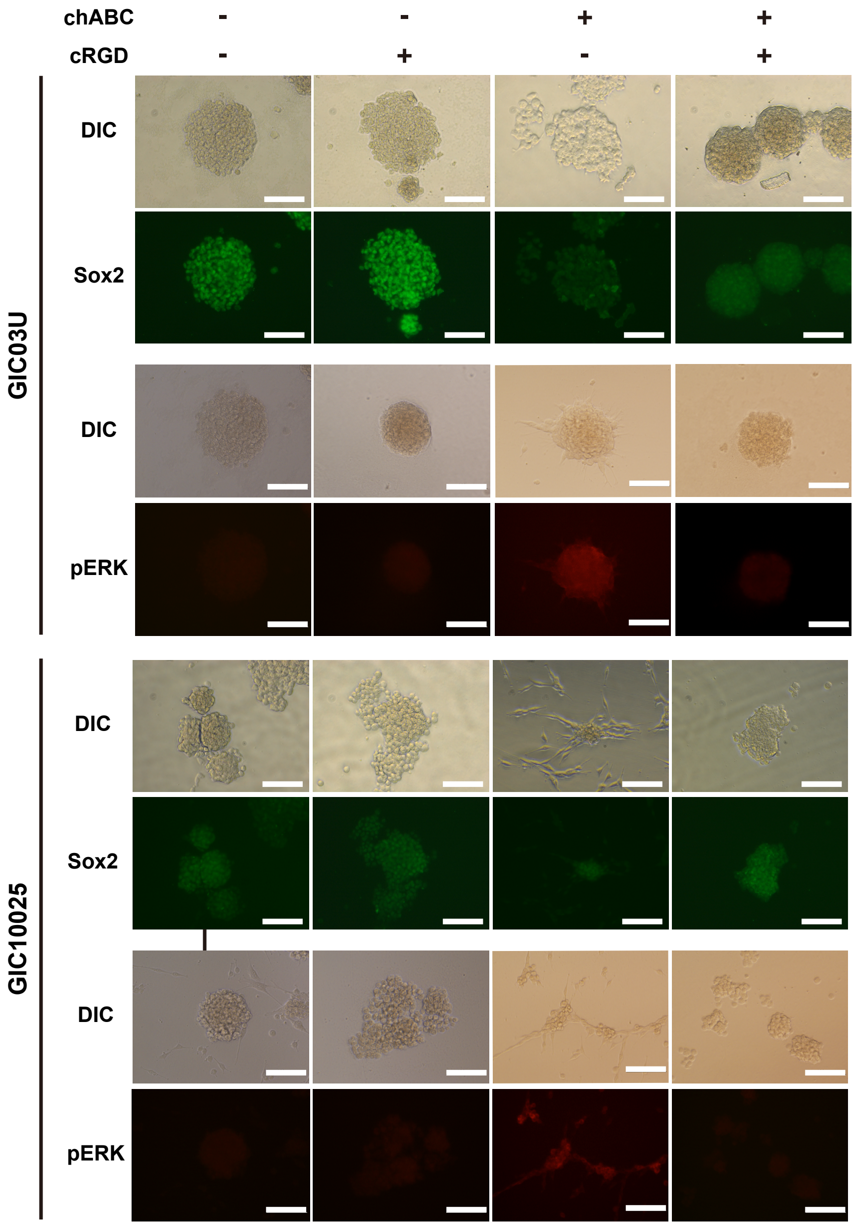

Figure S5. cRGD inhibits GIC differentiation induced by chABC. Fluorescent immunostaining of GIC spheres and differentiating GICs induced by chABC, after treatment with or without cRGD peptide for 24 h. Cells were stained with antibodies against Sox2 stem cell marker (Alexa 488: green), and phosphorylated p44/42 MAPK (pERK) (Alexa 568:red). Scale bars, 100 μm.

Figure S6

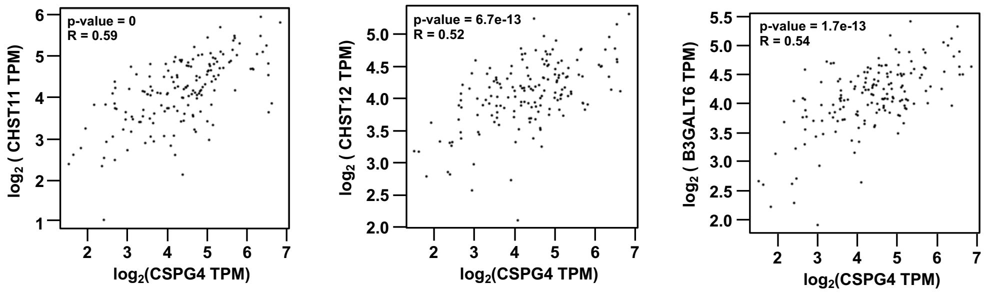

Figure S6. *In silico* analyses of the CSPG4 mRNA expression show the correlation with the chondroitin sulfate transferase mRNAs in GBMs. Correlations between CSPG4 and chondroitin sulfate transferases; CHST11, CHST12, and B3GALT6, in GBMs, analyzed by the GEPIA database.

Figure S7

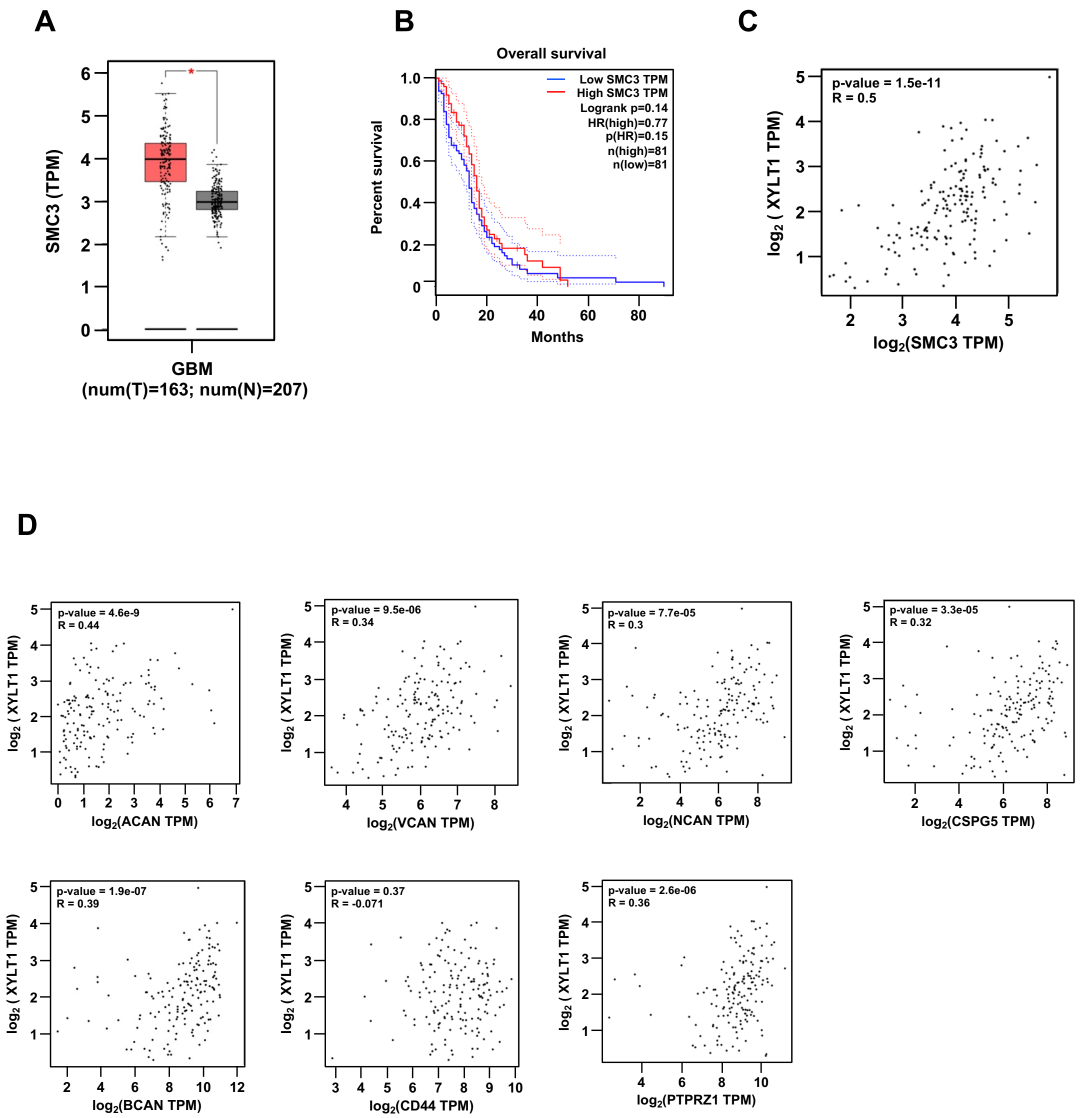

Figure S7. *In silico* analyses of the mRNA expression of eight CSPG families in GBMs. **(A)** SMC3 expression, **(B)** overall survival of GBM patients with high and low SMC3 expression, and **(C)** correlation between SMC3 and XYLT1 were analyzed by Gene Expression Profiling Interactive Analysis (GEPIA). Num (T) and num (N) in Figure S5A means the number of tumors and normal samples. **(D)** Correlation of other CSPG family, such as ACAN, VCAN, NCAN, CSPG5, BCAN, CD44, and PTPRZ1 with XYLT1 in GBMs.

Figure S8

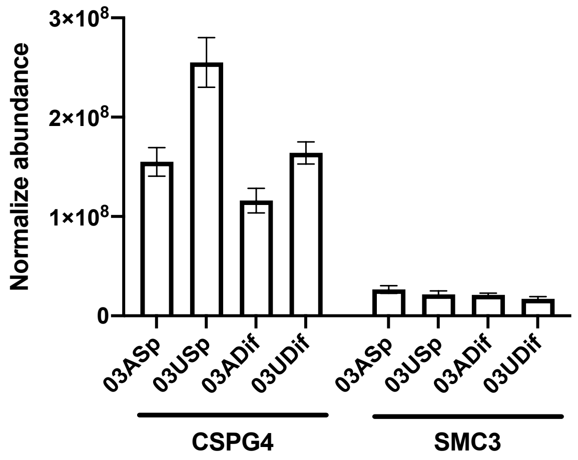

Figure S8. Comparison of CSPG4 and SMC3 protein expressions in GICs and differentiating GICs. Each protein expression was accessed with the MS abundances obtained from each sample global proteomics data (normalized average abundances of each fore replicates). The peptides identified from the MS data of CSPG4 and SMC3 from GIC03A and GIC03U cells after 48 hr of the incubation with serum to induce differentiations (Dif) or without serum to maintain sphere condition (Sp) were analyzed by the Label-Free MS Quantitation (nanoEASY-Orbitrap Fusion Tribrid system). CSPG4 Sp vs Dif : GIC03A ***p* = 0.0032, GIC03U ****p* = 0.00036, SMC3 Sp vs Dif :GIC03A **p* = 0.0268, GIC03U *p* = 0.0792, student *t-test.*

Figure S9

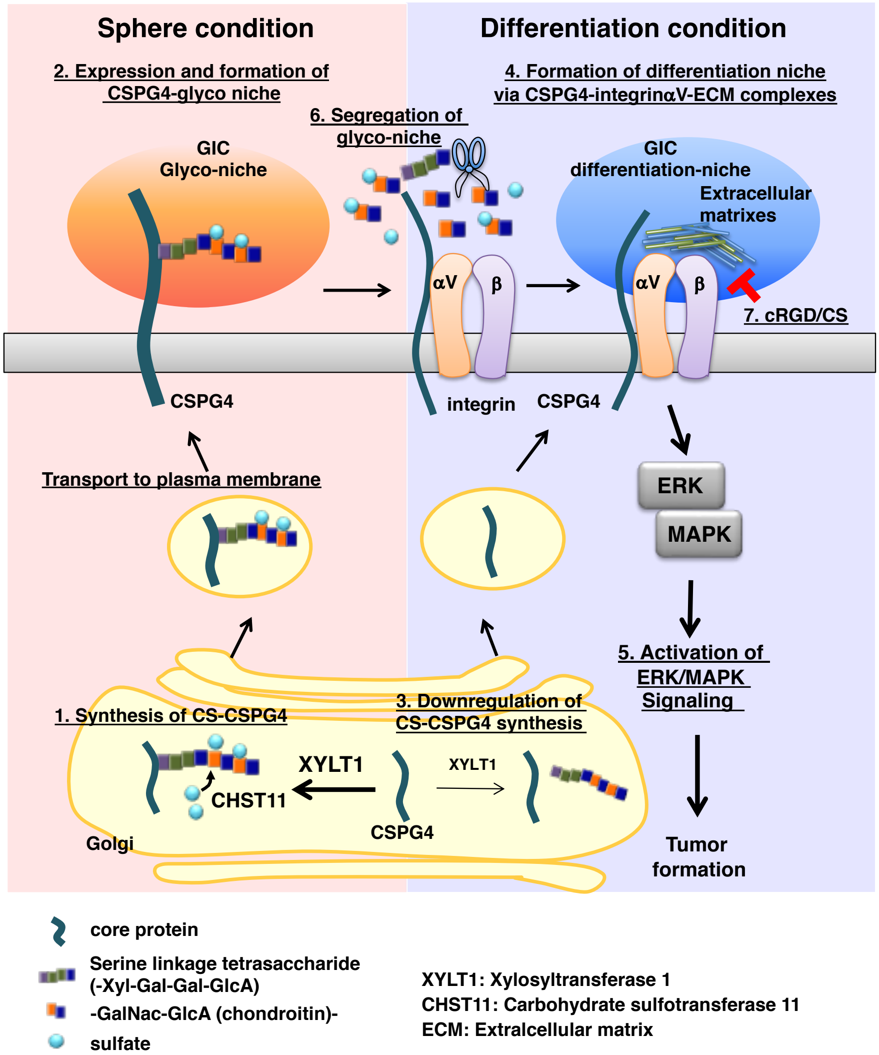

Figure S9. The postulated diagram of GIC differentiation mechanisms via a CSPG4-integrin αV-ERK/MAPK signaling pathway. **(1)** In the GIC sphere conditions, CS biosynthesis on CSPG4 is initiated by the transfer of a GalNAc residue to the linkage region of a GlcA-Gal-Gal-Xyl tetrasaccharide primer that is attached to a serine residue of the CSPG4 core protein (non-glycosylated) by XYLT1. XYLT1 and chondroitin 4-*O*-sulfo transferase 1 (CHST11) are activated and localized in the Golgi apparatus. **(2)** CS-CSPG4 is transported to the plasma membrane and expressed to form a 'CSPG4 associated glyco-niche' for maintenance of GIC stemness. **(3)** In the differentiation conditions, CS biosynthesis on CSPG4 is

downregulated with decreased expression of XYLT1 and CHST11, and thus low or non-glycosylated CSPG4 is transported to the plasma membrane. **(4)** After this CSPG4 transportation to the plasma membrane, it preferentially binds to integrin αV with the ECM to form the "differentiation niche". **(5)** ERK/MAPK signaling is upregulated via CSPG4-integrin αV-ECM complexes and induces GIC differentiation to promote tumor formation. **(6)** Manipulation of GIC glyco-niche degradation by chABC significantly induces GIC differentiation and upregulates ERK/MAPK signaling. **(7)** cRGD/CS administration suppresses the formation of the differentiation niche resulting in downregulation of ERK/MAPK signaling during GIC differentiation.
